# Supplementary material for: Assessment of allergy knowledge among the Palestinian community: A cross-sectional study
Source: PLoS One. 2025 Dec 17;20(12):e0339119. doi: 10.1371/journal.pone.0339119 (PMC12711017; doi:10.1371/journal.pone.0339119)
Supplement: S1 File — (PDF) [file pone.0339119.s001.pdf]

**Questionnaire:** The questions evaluate the level of knowledge about allergies.	
**1.Symptoms of anaphylaxis can occur:**	Short period of touching
	Long period of touching
	I don't know
	**Both are correct ***
**2.An anaphylactic reaction can be as simple as developing a rash after exposure to an allergen.**	Right
	**Wrong ***
	I don't know
**3.Anaphylaxis can occur from eating common foods such as milk, eggs, or shellfish.**	**Right ***
	Wrong
	I don't know
**4.Anaphylaxis always requires medical treatment.**	Right
	**Wrong ***
	I don't know
**5.The most severe form of allergic reaction is called anaphylaxis. Which symptoms might happen with this?**	Difficulty in Breathing
	Blood pressure decreasing (Hypotension)
	Rhinorrhea
	**All of the above ***

	I don't know
**6.If you are at risk for anaphylaxis, the best way to manage your condition is:**	Avoiding allergic materials
	Make a plan to manage allergic cases
	Always carry an epinephrine shot
	**All of the above ***
	I don't know
**7.Which of these body systems causes allergic reactions?**	**The immune system ***
	Endocrine
	Aautonomic nervous system
	I don't know
**8.An allergen is anything that triggers an allergic response. Which of these could be an allergen?**	Dust
	Food
	Nickel
	**All of the above ***
	I don't know
**9.Dust mites are a common trigger for indoor respiratory**	Curtains
	Carpet

**allergies. Where are you most likely to find them in the home?**	**Bed ***
	All of the above
	I don't know
**10.Allergies can cause conjunctivitis:**	**Right ***
	Wrong
	I don't know

Each question in the questionnaire was assigned one point for a correct answer and zero points for an incorrect answer, with a total possible score of 10. The scores were categorized into three levels of knowledge: weak level from (0) to (3), the average level was from (4) to (6), and a strong level from (7) to (10).
