# Supplementary material for: Assessment of allergy knowledge among the Palestinian community: A cross-sectional study
Source: PLoS One. 2025 Dec 17;20(12):e0339119. doi: 10.1371/journal.pone.0339119 (PMC12711017; doi:10.1371/journal.pone.0339119)
Supplement: Attachment — Submitted filename: Manuscript reviewed PLOS.docx [file pone.0339119.s003.docx]

Assessment of Allergy Knowledge Among the Palestinian Community: A Cross-Sectional Study.

1. In the abstract, start the second sentence with " adequate instead of proper
2. Spell check limited
3. Average, good, inadequate, insufficient, and weak were the terminologies used to describe or define the level of knowledge. Better to use either adequate and inadequate or sufficient and insufficient or good and poor or strong and weak to avoid confusion
4. Introduction well written
5. The study aims to estimate the prevalence, in addition to knowledge assessment, but not shown in the results
6. Justify the reasons for selecting 25 people for pilot study
7. Table 2 to be revised
