# Supplementary material for: Assessment of allergy knowledge among the Palestinian community: A cross-sectional study
Source: PLoS One. 2025 Dec 17;20(12):e0339119. doi: 10.1371/journal.pone.0339119 (PMC12711017; doi:10.1371/journal.pone.0339119)
Supplement: Attachment — Submitted filename: Allergy response letter 1-11-2025.docx [file pone.0339119.s005.docx]

Dear respected editor,

We would like to thank you and thank the reviewers for your valuable comments and suggestions which enhanced the quality of our manuscript. Kindly find below our responses and we highlighted these corrections in red in the revised manuscript.

Kind regards,

Muastafa Ghanim, Corresponding author

**Journal Requirements:**

When submitting your revision, we need you to address these additional requirements.

1. Please ensure that your manuscript meets PLOS ONE's style requirements, including those for file naming. The PLOS ONE style templates can be found at <https://journals.plos.org/plosone/s/file?id=wjVg/PLOSOne_formatting_sample_main_body.pdf> and <https://journals.plos.org/plosone/s/file?id=ba62/PLOSOne_formatting_sample_title_authors_affiliations.pdf>

Done.

2. We noticed you have some minor occurrence of overlapping text with the following previous publication(s), which needs to be addressed:

<https://pubmed.ncbi.nlm.nih.gov/39910132/>

In your revision ensure you cite all your sources (including your own works), and quote or rephrase any duplicated text outside the methods section. Further consideration is dependent on these concerns being addressed.

Any duplicated sentences were rephrased.

2. [Please include captions for your Supporting Information files at the end of your manuscript, and update any in-text citations to match accordingly. Please see our Supporting Information guidelines for more information: http://journals.plos.org/plosone/s/supporting-information.](http://track.editorialmanager.com/CL0/http:%2F%2Fjournals.plos.org%2Fplosone%2Fs%2Fsupporting-information./1/010f019a09c94e74-4400f279-1f4c-40cc-97b4-a8249f54de00-000000/OHLD2ifss-BT7NCFuV96Fe352EAUIDOKEALaI1NaMOo=234)

Done.

3. We note that there is identifying data in the Supporting Information file <Allergy Raw data.xlsx>. Due to the inclusion of these potentially identifying data, we have removed this file from your file inventory. Prior to sharing human research participant data, authors should consult with an ethics committee to ensure data are shared in accordance with participant consent and all applicable local laws.

   Data sharing should never compromise participant privacy. It is therefore not appropriate to publicly share personally identifiable data on human research participants. The following are examples of data that should not be shared:

   -Name, initials, physical address
   -Ages more specific than whole numbers
   -Internet protocol (IP) address
   -Specific dates (birth dates, death dates, examination dates, etc.)
   -Contact information such as phone number or email address
   -Location data
   -ID numbers that seem specific (long numbers, include initials, titled “Hospital ID”) rather than random (small numbers in numerical order)

   Data that are not directly identifying may also be inappropriate to share, as in combination they can become identifying. For example, data collected from a small group of participants, vulnerable populations, or private groups should not be shared if they involve indirect identifiers (such as sex, ethnicity, location, etc.) that may risk the identification of study participants.

   Additional guidance on preparing raw data for publication can be found in our Data Policy (<https://journals.plos.org/plosone/s/data-availability#loc-human-research-participant-data-and-other-sensitive-data>) and in the following article: <http://www.bmj.com/content/340/bmj.c181.long>.

   Please remove or anonymize all personal information (<specific identifying information in file to be removed>), ensure that the data shared are in accordance with participant consent, and re-upload a fully anonymized data set. Please note that spreadsheet columns with personal information must be removed and not hidden as all hidden columns will appear in the published file.

Done.

4. If the reviewer comments include a recommendation to cite specific previously published works, please review and evaluate these publications to determine whether they are relevant and should be cited. There is no requirement to cite these works unless the editor has indicated otherwise.

Done.

6. Please review your reference list to ensure that it is complete and correct. If you have cited papers that have been retracted, please include the rationale for doing so in the manuscript text, or remove these references and replace them with relevant current references. Any changes to the reference list should be mentioned in the rebuttal letter that accompanies your revised manuscript. If you need to cite a retracted article, indicate the article’s retracted status in the References list and also include a citation and full reference for the retraction notice.

The reference list was reviewed and any errors were corrected.

**Reviewer #1:**

1. In the abstract, start the second sentence with " adequate instead of proper

Response: Correction is done.

2. Spell check limited

Response: Thank you for your comment. The text of all of the manuscript was reviewed by authors for any typo and linguistic errors. Also, the manuscript was edited by our colleague Dr. Waleed Salameh, an expert in Educational English from the Faculty of Graduate Studies at An-Najah National University, for whom we added acknowledgement at the end of the manuscript.

3. Average, good, inadequate, insufficient, and weak were the terminologies used to describe or define the level of knowledge. Better to use either adequate and inadequate or sufficient and insufficient or good and poor or strong and weak to avoid confusion

Response: Thank you for your comment. The correction was done. The words: strong, average, and weak are used throughout the manuscript to describe the knowledge levels to make it uniform and prevent confusion. The knowledge levels are divided in the manuscript into three levels based on the study tool description.

4. Introduction well written

Thanks for your support.

5. The study aims to estimate the prevalence, in addition to knowledge assessment, but not shown in the results

Response: Thank you for your notice. The study aimed to evaluate the knowledge about allergies among Palestinians. We have noticed that prevalence is used once at the end of the introduction, and proper correction is done.

6. Justify the reasons for selecting 25 people for pilot study.

Response: The sample size of 25 participants was selected based on established recommendations for pilot studies, where sample sizes of anywhere from 12 to 35 have been proposed, and it is considered sufficient for estimating parameters in questionnaire-based studies. Proper justification with appropriate references has been added to the methods section in the manuscript.

7. Table 2 to be revised.

Response: Table 2 is revised.

**Reviewer #2:**

I congratulate you for this important work. This study assessed allergy-related knowledge among 1,002 Palestinians aged 18 years and older using a validated online questionnaire. The mean knowledge score was 5.4/10, with most participants demonstrating average awareness of allergy causes and symptoms but notable gaps in understanding management strategies, such as the use of epinephrine and recognition of dust mites as a major allergen. Knowledge levels varied significantly by gender, education, occupation, and prior personal or social experience with allergy. The authors conclude that while general awareness is fair, targeted educational campaigns are needed to address critical misconceptions and improve community preparedness for allergy-related emergencies. some points for improvment:

1. The sample is skewed toward younger and well-educated participants. Please expand discussion on how this limits generalizability, particularly for older, rural, or less literate populations. Highlight how online distribution might exclude vulnerable groups most at risk of poor allergy awareness.

Response: Correction is done, the discussion has been expanded to acknowledge the limitations in sample representativeness.

2. The finding that only 4% recognized dust mites as a common allergen and that just over half understood anaphylaxis management is critical. These points should be emphasized further as priorities for targeted public health education. Consider linking these gaps to regional healthcare challenges, e.g., access to epinephrine autoinjectors.

Response: Correction is done.

3. While p-values are reported, consider also including effect sizes or confidence intervals to quantify differences more meaningfully.

Response: Thank you for your suggestion. The 95% confidence intervals of the difference are added to Table 4.

4. The IRB approval and informed consent are clearly reported. It might be helpful to briefly state in the discussion how ethical safeguards strengthened participant trust and response quality.

Done.
